# JCPyV miR-J1-5p in Urine of Natalizumab-Treated Multiple Sclerosis Patients

**DOI:** 10.3390/v13030468

**Published:** 2021-03-12

**Authors:** Simone Agostini, Roberta Mancuso, Andrea Saul Costa, Domenico Caputo, Mario Clerici

**Affiliations:** 1 IRCCS Fondazione Don Carlo Gnocchi ONLUS, 20148 Milan, Italy; rmancuso@dongnocchi.it (R.M.); acosta@dongnocchi.it (A.S.C.); dcaputo@dongnocchi.it (D.C.); mario.clerici@unimi.it (M.C.); 2 Department of Pathophysiology and Transplantation, University of Milan, 20122 Milan, Italy

**Keywords:** multiple sclerosis, JCPyV, miRNA, rehabilitation, biomarker

## Abstract

The use of Natalizumab in Multiple Sclerosis (MS) can cause the reactivation of the polyomavirus JC (JCPyV); this may result in the development of progressive multifocal leukoencephalopathy (PML), a rare and usually lethal disease. JCPyV infection is highly prevalent in worldwide population, but the detection of anti-JCPyV antibodies is not sufficient to identify JCPyV infection, as PML can develop even in patients with negative JCPyV serology. Better comprehension of the JCPyV biology could allow a better understanding of JCPyV infection and reactivation, possibly reducing the risk of developing PML. Here, we investigated whether JCPyV miR-J1-5p—a miRNA that down-regulates the early phase viral protein T-antigen and promotes viral latency—could be detected and quantified by digital droplet PCR (ddPCR) in urine of 25 Natalizumab-treated MS patients. A 24-month study was designed: baseline, before the first dose of Natalizumab, and after 1 (T1), 12 (T12) and 24 months (T24) of therapy. miR-J1-5p was detected in urine of 7/25 MS patients (28%); detection was possible in three cases at T24, in two cases at T12, in one case at T1 and T12, and in the last case at baseline and T1. Two of these patients were seronegative for JCPyV Ab, and viral DNA was never found in either urine or blood. To note, only in one case miR-J1-5p was detected before initiation of Natalizumab. These results suggest that the measurement of miR-J1-5p in urine, could be a biomarker to monitor JCPyV infection and to better identify the possible risk of developing PML in Natalizumab-treated MS patients.

## 1. Introduction

The Polyomavirus JC (JCPyV), isolated for the first time in 1971 [1], is a ubiquitous human neurotropic virus belonging the family Polyomaviridiae. The genome of JCPyV is a circular non-enveloped double-strand DNA [2] and it is divided into early and late genes, separated by a non-coding control region (NCCR) that contains the origin of DNA replication (ori) as well as the promoter and the enhancer elements [3]. MicroRNAs (miRNAs) are short non-coding single-strand RNAs (about 20–24 nucleotides) involved in mRNA silencing and post-transcriptional regulation of gene expression. Mature miRNAs are generated through the two-step cleavage of primary miRNA (pri-miRNA): the first cut takes place inside the nucleus, where pri-miRNAs become precursor miRNAs (pre-miRNAs); the second cut takes place into cytoplasm and give rise to the mature miRNAs [4].

Even the viruses are able to express miRNAs, and regarding the polyomaviruses they are discovered for the first time in simian virus 40 (SV40) [5]. The miRNAs of human polyomaviruses (SV40, JCPyV and BKPyV) show a partial, shared sequence identity. Their function is not yet completely understood, but they seem to have both viral and host mRNA targets, escaping host immune response [4,5,6,7].

**Figure 1:**
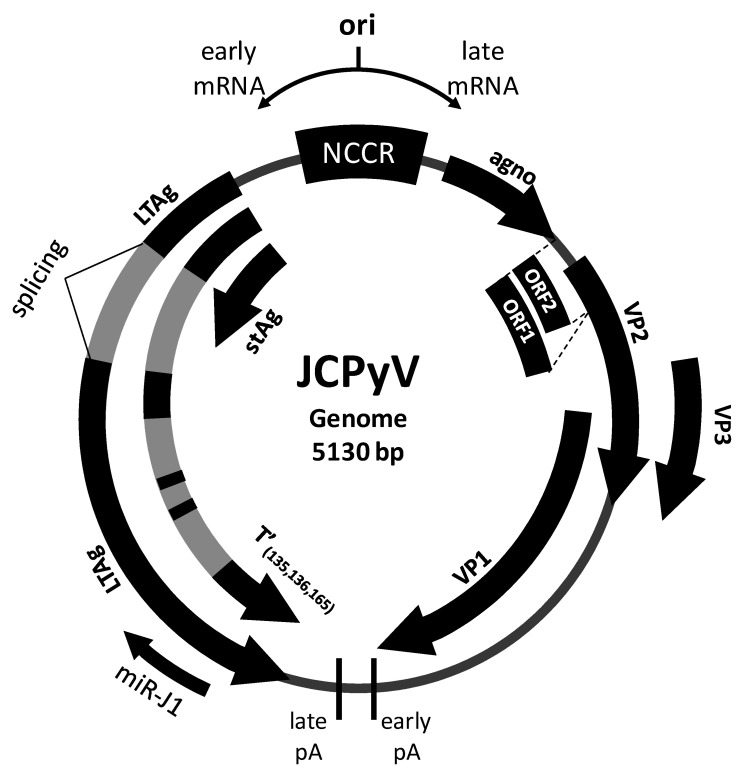
Genomic organization of the polyomavirus JC (JCPyV). ori: origin of replication region; NCCR: non-coding control region; LTAg: large T-Antigen; sTAg: small T-antigen; T’_(135,136,165)_: T-antigen splice variants [15,16]; VP1-3: major capsid proteins; Agno: Agnoprotein (agno); pA: polyadenylation site; ORF: open reading frame [17,18].

JCPyV expresses a pri-miRNA (pri-miR-J1) that, is processed by Drosha-DGCR8 microprocessor [8], in a pre-miRNA (pre-miRJ-1). After the export into cytoplasm this pre-miRNA is cleaved into two mature miRNAs: miR-J1-5p and miR-J1-3p [9,10,11,12] (Figure 1). The role and function of these two miRNAs are not completely known, but it is suggested that they can repress JCPyV replication [10,13] Moreover they may downregulate the host immune response, either directly, via the modulation the expression of cellular genes involved in host immune responses [14], or indirectly, reducing the expression of the viral T antigen [12].

Primary JCPyV infection is usually asymptomatic and is observed early in life; the virus subsequently establishes a life-long latent infection in different host cells, and in particular in proximal renal tubules and mononuclear blood cells [19]. Generally, JCPyV infection is completely asymptomatic [20]. However, in some rare cases, mainly in immunosuppressed subjects, the virus can enter in a lytic phase in oligodendrocytes and can cause progressive multifocal leukoencephalopathy (PML), a severe, and often fatal, demyelinating disease characterized by the presence of inclusion bodies in the nuclei of infected cells [21,22]. Because immunosuppression facilitates JCPyV reactivation, the use of monoclonal antibody (mAB)-based therapies targeting immune molecules is considered to be a risk-factor for PML.

Natalizumab, in particular, is a humanized anti-α4 integrin mAB that binds the α4 subunit of the α4β1 and α4β7 integrin chains of very late antigen-4 (VLA-4), a protein that mediates cell migration through its binding to the vascular cell adhesion molecule (VCAM). As a consequence, leukocytes migration and extravasation are impaired [23]. As one of the hallmarks of Multiple Sclerosis (MS) is chronic leukocyte infiltration in the brain, Natalizumab is used for this disease. Natalizumab was firstly approved by the US Food and Drug Administration (FDA) in 2004 for relapsing remitting MS (RRMS). Its effects were clearly evident, as several studies showed that the rate of Expanded Disability Status Scale (EDSS) worsening was slowed down by natalizumab [24,25,26,27,28]. Natalizumab however was observed to be plagued by a rare but potentially lethal adverse effect. Thus, blocking the infiltration of immune cells into the central nervous system (CNS) hampers immune surveillance, possibly allowing JCPyV reactivation and the development of PML [28]. The risk of developing PML increases if therapy lasts for more than two years and with previous use of immunosuppressant therapies [29]. Recent data indicate that the overall incidence of PML in natalizumab-treated patients is 4.16 per 1000 [30].

The presence of JCPyV-specific antibodies is another important risk factor for PML [28,31] but, on the other hand, patients with negative JCPyV serology can develop PML [32,33]. JCPyV-specific antibodies thus are not sufficient for the diagnosis of JCPyV infection [32]. This whole issue is further complicated by the observation that miR-J1-5p and -3p are expressed in PML, but they can also be observed in MS patients without PML and even in healthy controls [34,35]. To note, the miR-J1-3p shares its sequence with miR-B1-3p [13,14], a miRNA expressed by the BKPyV polyomavirus [36], whereas miR-J1-5p sequence is restricted to JCPyV.

In the attempt to identify more reliable markers for JCPyV infection and reactivation, we monitored miR-J1-5p expression—an unambiguous signal of infection compared to antibodies—in urine of JCV infected RRMS patients treated with Natalizumab for a 24-month period.

## 2. Materials and Methods

### 2.1. Study Population and Specimens

A total of twenty-five patients with a diagnosis of RRMS, according to McDonald criteria [37], were enrolled in the study. The patients, followed by the Multiple Sclerosis Unit of IRCCS Santa Maria Nascente, Fondazione Don Gnocchi, Milan, Italy, and enrolled in a rehabilitation program, were treated with Natalizumab, and had been previously immunologically and virologically characterized [38,39]. The JCPyV serostatus and the presence of JCPyV DNA in biological samples (urine, serum, blood and cerebrospinal fluid) was validated in all patients. All patients fulfilled the Italian Agency of Drug (AIFA) criteria for Natalizumab treatment, i.e., they either were affected by a particularly severe disease course in the year prior to therapy (marked clinical worsening; high relapse rate; rapid disability accumulation), or they showed a lack of response to previous immunosuppressive or immunomodulatory therapies. The washout period before initiating Natalizumab was 3 months for patients previously using immune-modifying drugs, and 6 months for patients undergoing immunosuppressive therapy (mandatory according to AIFA guidelines). Routine brain magnetic resonance (MRI) was obtained at both baseline and after 1 and 2 years of treatment, and results were reviewed by local radiologists.

Urine were collected from all the individuals enrolled in the study at baseline and after 1 (Month 1), 12 (Month 12) and 24 (Month 24) months of therapy, at the time of Natalizumab infusion.

The study conformed to the ethical principles of the Declaration of Helsinki; all enrolled subjects gave informed and written consent according to a protocol approved by the local ethics committee (approved on 28/11/2012).

### 2.2. miRNA Extraction from Urine and cDNA Reverse Transcription

miRNA isolation from urines was performed with a column-based kit (MiRNeasy serum/plasma Kit, Qiagen GmbH, Hilden, Germany), according to specific protocol. Total RNA was transcribed in cDNA (3 ng) using a Universal cDNA synthesis kit II (Qiagen GmbH, Hilden, Germany), according to the following protocol: 60 min at 42 °C, followed by heat-inactivation of the reverse transcriptase for 5 min at 95 °C.

### 2.3. Digital Droplet PCR

miR-J1-5p detection was performed by digital droplet PCR (ddPCR QX200, Bio-Rad, Hercules, CA, US). The ddPCR assays were optimized to obtain an optimal separation between positive and negative droplets using, serial dilutions of RNA, different primer concentrations, and annealing temperatures.

For miR-J1-5p detection, 3 μL of cDNA (1:25) was mixed with LNA^TM^-miR-J1-5p primers (Qiagen GmbH, Hilden, Germany), and ddPCR EvaGreen Supermix (Bio-Rad), which was then emulsified with droplet generator oil (Bio-Rad Hercules, CA, US) using a QX200 droplet generator according to the manufacturer’s instruction. The droplets were then transferred to a 96-well reaction plate and heat-sealed with a pierceable sealing foil sheet (PX1, PCR plate sealer, Bio-Rad Hercules, CA, US). The PCR amplification was performed in sealed 96-well plate using a T100 thermal cycler (Bio-Rad Hercules, CA, US) as follows: 10 min at 95 °C, 40 cycles consisting of a 30-s denaturation at 94 °C and a 60-s annealing and extension at 59 °C, followed by 5 min at 4 °C, 5 min at 90 °C and a hold at 4 °C. Following PCR amplification, the 96-well plate was transferred to a QX200 droplet reader (Bio-Rad Hercules, CA, US). Each well was queried for fluorescence to determine the quantity of positive events (droplets), and the results were displayed as dot plots.

### 2.4. Data Analysis

For ddPCR analysis, the QuantaSoft software version 1.7.4.0917 (Bio-Rad Hercules, CA, US) was used to quantify copies of miR-J1-5p. Thresholds were determined manually for each experiment, according to the negative controls, which included a no template control. Droplet positivity was determined by fluorescence intensity; only droplets above a minimum amplitude threshold were counted as positive. Positive controls as well as negative controls were included in each experiment. Samples resulted in less than two positive droplets are considered negative [40]. The detection limit is 0.8 copies/μL of reaction. To assess the linearity and the accuracy of ddPCR at different target concentration, we ran the assay in triplicate on serial dilutions of miR-J1-5p cDNA in water. We found an extremely high concordance among series, and the responses were highly linear over the dynamic range of five orders to magnitude (R^2^ = 0.9998 and *p* ≤ 0.0001) [41]. Normally distributed data were summarized as mean and standard deviation (SD), whereas not normally distributed data were summarized as median and interquartile range (IQR: 25th and 75th percentile).

## 3. Results

### 3.1. Demographic, Clinical Characteristics and Virological Data of the Subjects

Therapy was well tolerated by all 25 RRMS patients (20 females and 5 males, mean age: 36.12 ± 8.60 years). Clinical relapses during the follow-up period were observed in 7/25 patients and the EDSS was not increased after the 24 months of treatment (before treatment: 4.0 ± 1.5; after treatment: 3.8 ± 1.6). Before receiving Natalizumab, fifteen patients (60%) had been treated with disease modifying therapies and 7 (28%) underwent immunosuppressive therapies. Eight patients (32%) showed MRI activity during the first year of treatment, and 2 (8%) during the second year. Importantly, no cases of PML were detected during the study period.

Results on JCPyV seropositivity and the presence of JCPyV DNA in blood and urine during the 24-months study period are summarized in Table 1 and in Supporting Information Table S1. The serostatus was evaluated in serum sample collected at the end of the two years of Natalizumab treatment. On the whole, based on the presence of antibodies and/or JCPyV DNA, 16 patients were considered as JCPyV infected and 9 as uninfected. Amongst the 16 JCPyV infected individuals, reactivation during therapy (i.e., presence of viral DNA in urine/blood at least once during treatment) was observed in 11 cases (from patient MS6 to patient MS16), whereas viral latency, both in urine and in blood, characterized 5 patients (from patient MS1 to patient MS5).

### 3.2. miR-J1-5p Expression in Urine

miR-J1-5p was detected in urine of 7/25 (28%) patients during the 24-months study period. In one case miR-J1-5p was detected even before the initiation of treatment, whereas in another case miR-J1-5p was found at two different time-points during treatment. JCPyV DNA was detected at least once in urine during the study period in six of the seven miR-J1-5p-positive patients; 4 of these patients were also JCPyV seropositive, 1 was seronegative (patient MS16) and in 1 JCPyV serostatus was not measured, but viral DNA was found in biological samples.

**Figure 2:**
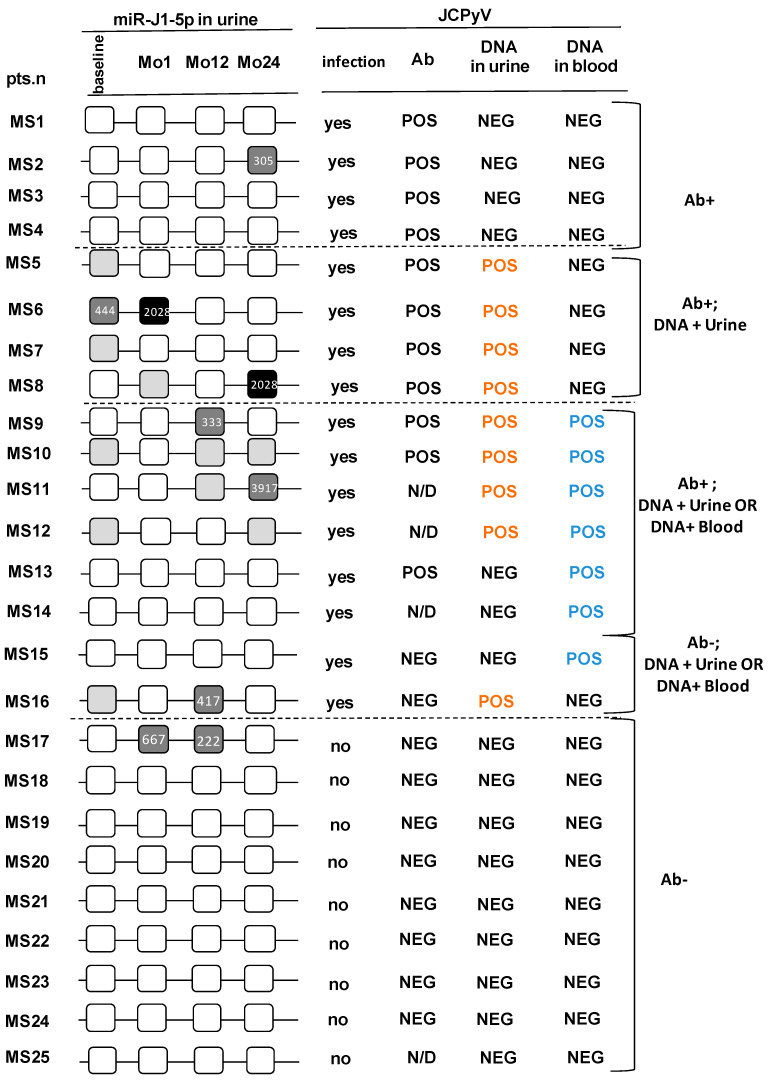
miR-J1-5p and JCPyV DNA detection in urine samples of Natalizumab-treated Multiple Sclerosis patients. Black boxes: positivity for both miR-J1-5p and JCPyV DNA; dark grey boxes: miR-J1-5p positivity alone; light grey boxes: JCPyV DNA positivity alone; white boxes: absence of both nmiR-J1-5p and JCPyV DNA. miR-J1-5p copies/ng are indicated inside the boxes. In the right columns the presence of anti-JCPyV Antibodies (Ab) and JCPyV DNA for each patient is shown. For the exact timing of DNA viral detection in urine and blood, please see Table S1. Mo: Month; pts.n: patient number; N/D: not defined; MS: Multiple Sclerosis; POS: positive; NEG: negative.

Of the 18 patients in whom miR-J1-5p could not be detected, 7 were JCPyV seropositive and 7 were JCPyV seronegative. One additional patient was JCPyV seronegative, but viral DNA could be isolated from blood. JCPyV serostatus was not measured in the remaining 3 miR-J1-5p-negative patients, in two of whom, nevertheless viral DNA was found in blood. These results are summarized in Figure 2, that includes the miR-J1-5p quantitation for each positive sample (copies/ng) as well.

Analyses performed upon dividing up patients in those who were (*n* = 16) or were not (*n* = 9) JCPyV-infected showed that miR-J1-5p was detected in urine of 6 of the 16 JCPyV-infected patients. Viral DNA was detected as well in urine and/or blood in 5 of these cases, indicating the presence of viral reactivation. JCPyV- DNA was never detected in the fifth patient, suggesting the persistence of viral latency. Surprisingly, miR-J1-5p was detected as well in the urine of 1 (MS17) of the 9 JCPyV-uninfected patients (i.e., individual who was always JCPyV- seronegative and in whom viral DNA could never be detected) (Figure 2). Possible correlation between age/gender and virological data were examined; no such correlation could be observed.

## 4. Discussion

When the clinician has to evaluate the best treatment for a patient with MS, JCPyV infection must be considered, as several drugs—Natalizumab, Fingolimod, Dimethyl fumarate—are known to increase the risk of developing PML, a disease caused by the reactivation of JCPyV [30,42,43,44]. For this reason, a sensitive and reliable method to screen and monitor for JCPyV infection is extremely important in the setting of therapy for MS patients. JCPyV infection is evaluated by measuring virus-specific antibodies (Ab) in serum/plasma. However, it is important to underline that false negative results are frequent: Berger and coworkers found that the false-negative rate is about 37% [32], and recently PML cases have been reported in JCPyV seronegative MS patients [33]. Detection of viral DNA can be also performed by qPCR in different biological fluids (blood, urine, CSF), to assess the infection in seronegative subjects and to evidence viral reactivation; notably, though, even in patients with a diagnosis of PML JCPyV viral load can be very low or even undetectable [45].

microRNAs are gaining a growing importance as fundamental biomarkers in the diagnosis and the follow-up of treatments or chronic pathologies, including MS [46,47,48]. JCPyV miR-J1-5p, in particular, was found to be present in extremely high concentration in brain tissue of PML patients [13] suggesting that this miRNA is potentially a biomarker for PML. For this reason, in the present work we measured the expression of the JCPyV miR-J1-5p in urine of 25 MS patients before and during 24 months of Natalizumab treatment; results herein show that JCPyV miR-J1-5p is present in urine of a minority (28%) of patients. Notably, the percentage of miR-J1-5p-positive MS patients was lower than that observed in healthy controls (62%) [34]. This difference is quite surprising, as we did not find an association among miR-J1-5p and viral reactivation: other studies are therefore necessary to clarify this aspect. To note, the urine used for the present study were collected and stored, in some cases for some years, at –70°C prior the miRNAs extraction. Although these samples were never thawed, and it is known that miRNAs are quite stable in biological fluids [49], including in urine [50], we cannot rule out that the relative low frequency of positivity we observed is due to the sampling timing.

miR-J1-5p was detectable not only in JCPyV seropositive (25%) but also in two JCPyV seronegative patients. Notably, in one of these miR-J1-5p-positive/JCPyV seronegative patients (MS17), viral DNA could not be detected either in blood or urine. This is an intriguing result: as miR-J1-5p seems to be more expressed in latent viral infection [34], a possible explanation is that in this patient the viral load in biological fluids was so low to be undetectable with standard methods.

These findings confirm previous observations [35] showing that JCPyV infection rate can be higher than that expected from serology alone. These results also suggest that miR-J1-5p measurement can be a useful support for identification of MS patients at risk to develop PML prior to disease modify treatment (DMT). It is important to underline that, even if fortunately no cases of PML were observed in this cohort of patients, in all but one case miR-J1-5p was detected in urine after the initiation of Natalizumab. This result confirms that the use of DMTs does facilitate the reactivation of JCPyV replication; this might not result in the development of PML possibly because it is of limited intensity, or because of the absence of yet undefined cofactors.

miR-J1-5p had been analyzed in urine [34,51], and previous results showed that miR-J1-5p could be detected in urine and plasma of both JCPyV-seropositive and -seronegative MS patients treated with Natalizumab [52]. However, it is important to underline that in this case miRNAs extraction was performed from exosomes and not directly from urine. The presence of circulatory JCPyV miRNAs in JCPyV-seronegative MS patients was also confirmed in a recent work on patients treated with Natalizumab and interferon-beta, in whom miR-J1-5p was measured directly from plasma [53]. In that cross-sectional work miR-J1-5p was detected in 78% of JCPyV seronegative MS patients—and in 84% of patients treated with Natalizumab. Our results for the first time monitor miR-J1-5p expression in urine at different time-points during 24 months of Natalizumab treatment, and, importantly, show how this miRNA can be measured in whole urine: a very easy and not-invasive procedure. In conclusion, our results show that miR-J1-5p is detectable in human urine and confirm that JCPyV serostatus actually underestimates the true JCPyV infection rate. The measurement of miR-J1-5p in urine, together with the evaluation of viral load and of serostatus, can give a more complete picture of JCPyV infection and reactivation. The importance of combination of JCPyV serostatus and viral load in evaluating the risk of PML in MS notwithstanding, the results herein suggest that analyzing miR-J1-5p in biological fluids could offer some diagnostic advantages. Further studies on larger groups of subjects will need to clarify the meaning of fluctuation of expression of this miRNA and their impact of viral replication.

## Figures and Tables

**Table 1:** JCPyV characterization in the enrolled patients.

	*n*	JCPyV Seropositivity	JCPyV Viremia	JCPyV Viruria	miR-J1-5p Detection in Urine
JCPyV infected patients	16	11/16 (68.7%)	7/16 (43.8%)	9/16 (56.2%)	6/16 (37.5%)
JCPyV uninfected patients	9	0/9 (0.0%)	0/9 (0.0%)	0/9 (0.0%)	1/9 (11.1%)

*n*: number of enrolled subjects

## Data Availability

The data presented in this study are available on request from the corresponding author.

## Supplementary Materials

The following are available online at https://www.mdpi.com/1999-4915/13/3/468/s1, Table S1: JCPyV seropostivity, viruria and viremia during the 24-months study period for each enrolled subject.
