# Supplementary material for: JCPyV miR-J1-5p in Urine of Natalizumab-Treated Multiple Sclerosis Patients
Source: Viruses. 2021 Mar 12;13(3):468. doi: 10.3390/v13030468 (PMC8000901; doi:10.3390/v13030468)
Supplement: Supplementary file 1 [file viruses-13-00468-s001.pdf]

Table S1. JCPyV seropostivity, viruria and viremia during the 24-months study period for each enrolled subject.

Patient	miRj1-5p in urine				JCPyV Viruria					JCPyV Viremia					JCPyV Ab
**Month**	baseline	Mo1	Mo12	Mo24	baseline	Mo1	Mo6	Mo12	Mo24	baseline	Mo1	Mo6	Mo12	Mo24	Post Mo24
MS1	NEG	NEG	NEG	NEG	NEG	NEG	NEG	NEG	NEG	NEG	NEG	NEG	NEG	NEG	POS
MS2	NEG	NEG	NEG	POS	NEG	NEG	NEG	NEG	NEG	NEG	NEG	NEG	NEG	NEG	POS
MS3	NEG	NEG	NEG	NEG	NEG	NEG	NEG	NEG	NEG	NEG	NEG	NEG	NEG	NEG	POS
MS4	NEG	NEG	NEG	NEG	NEG	NEG	NEG	NEG	NEG	NEG	NEG	NEG	NEG	NEG	POS
MS5	NEG	NEG	NEG	NEG	POS	NEG	NEG	NEG	NEG	NEG	NEG	NEG	NEG	NEG	POS
MS6	POS	POS	NEG	NEG	POS	POS	NEG	NEG	NEG	NEG	NEG	NEG	NEG	NEG	POS
MS7	NEG	NEG	NEG	NEG	POS	NEG	POS	NEG	NEG	NEG	NEG	NEG	NEG	NEG	POS
MS8	NEG	NEG	NEG	POS	NEG	POS	NEG	NEG	POS	NEG	NEG	NEG	NEG	NEG	POS
MS9	NEG	NEG	POS	NEG	NEG	NEG	NEG	POS	NEG	NEG	NEG	NEG	POS	NEG	POS
MS10	NEG	NEG	NEG	NEG	POS	NEG	NEG	POS	POS	POS	NEG	POS	NEG	NEG	POS
MS11	NEG	NEG	NEG	POS	NEG	NEG	POS	NEG	POS	NEG	POS	NEG	NEG	NEG	N/D
MS12	NEG	NEG	NEG	NEG	POS	NEG	NEG	NEG	POS	NEG	POS	NEG	NEG	NEG	N/D
MS13	NEG	NEG	NEG	NEG	NEG	NEG	NEG	NEG	NEG	NEG	NEG	NEG	NEG	POS	POS
MS14	NEG	NEG	NEG	NEG	NEG	NEG	NEG	NEG	NEG	NEG	NEG	NEG	POS	NEG	N/D
MS15	NEG	NEG	NEG	NEG	NEG	NEG	NEG	NEG	NEG	POS	NEG	NEG	NEG	NEG	NEG
MS16	NEG	NEG	POS	NEG	POS	NEG	POS	NEG	NEG	NEG	NEG	NEG	NEG	NEG	NEG
MS17	NEG	POS	POS	NEG	NEG	NEG	NEG	NEG	NEG	NEG	NEG	NEG	NEG	NEG	NEG
MS18	NEG	NEG	NEG	NEG	NEG	NEG	NEG	NEG	NEG	NEG	NEG	NEG	NEG	NEG	NEG
MS19	NEG	NEG	NEG	NEG	NEG	NEG	NEG	NEG	NEG	NEG	NEG	NEG	NEG	NEG	NEG
MS20	NEG	NEG	NEG	NEG	NEG	NEG	NEG	NEG	NEG	NEG	NEG	NEG	NEG	NEG	NEG
MS21	NEG	NEG	NEG	NEG	NEG	NEG	NEG	NEG	NEG	NEG	NEG	NEG	NEG	NEG	NEG
MS22	NEG	NEG	NEG	NEG	NEG	NEG	NEG	NEG	NEG	NEG	NEG	NEG	NEG	NEG	NEG
MS23	NEG	NEG	NEG	NEG	NEG	NEG	NEG	NEG	NEG	NEG	NEG	NEG	NEG	NEG	NEG
MS24	NEG	NEG	NEG	NEG	NEG	NEG	NEG	NEG	NEG	NEG	NEG	NEG	NEG	NEG	NEG
MS25	NEG	NEG	NEG	NEG	NEG	NEG	NEG	NEG	NEG	NEG	NEG	NEG	NEG	NEG	N/D

Ab: Antibody; Mo: Month; MS: Multiple Sclerosis; POS: positive; NEG: negative; N/D: not defined
